# Will long-read sequencing technologies replace short-read sequencing technologies in the next 10 years?

**DOI:** 10.4102/ajlm.v9i1.1340

**Published:** 2020-11-26

**Authors:** Boluwatife A. Adewale

**Affiliations:** 1 Medicine and Surgery, Faculty of Clinical Sciences, College of Medicine, University of Ibadan, Ibadan, Nigeria; 2 College Research and Innovation Hub (CRIH), University of Ibadan, Ibadan, Nigeria; 3 University College Hospital, Ibadan, Nigeria

The year 1977 is quite remarkable in the history of genomics. It was the first time that the complete genome of an organism (phage φX174) would be sequenced – the advent of the first generation of sequencing technologies. Of the two sequencing methods published that year, Fred Sanger’s ‘chain-termination’ method would become the mainstay of sequencing technology for the next three decades. Because of its better usability compared to the Maxam-Gilbert method, it was widely preferred and became commercialised^1^ by Applied Biosystems Inc. Thanks to the collaborative efforts of scientists across the world,^2^ Sanger sequencing eventually produced a reference human genome, courtesy of the US$2.7-billion (United States dollar) Human Genome Project completed in 2003.^3^

## Next-generation sequencing

The launch of pyrosequencing by 454 Life Sciences in 2005 marked the beginning of the second generation of sequencing. Massively parallel or next-generation sequencing (NGS) technologies eliminated the need for multiple personnel working on a genome by automating DNA cleavage, amplification and parallel short-read sequencing on a single instrument, thereby lowering costs and increasing throughput.^1,4^

**FIGURE 1:**
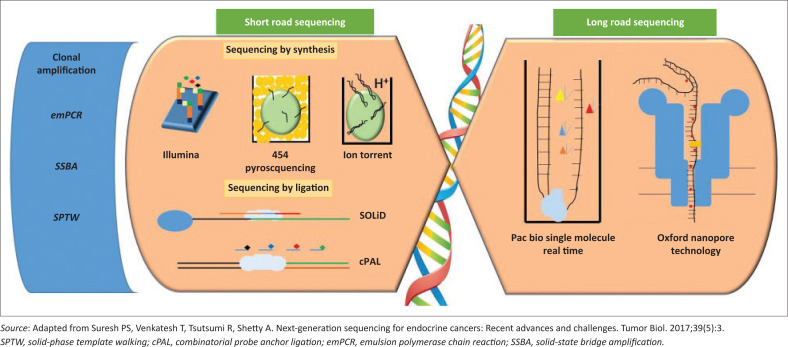
Overview of short- and long-read sequencing technologies. Short-read sequencing methods are displayed on the left, long-read sequencing methods are shown on the right. Short-read sequencing methods are classified into sequencing by synthesis and sequencing by ligation. They all require clonal amplification; emulsion polymerase chain reaction for 454 pyrosequencing, SOLiD™ and Ion Torrent, SSBA is peculiar to Illumina, solid-phase template walking is used for certain SOLiD™ technologies. Illumina, fluorescent-labelled dNTP added to bridge amplified DNA template; 454 pyrosequencing, empolymerase chain reaction-generated microbead-bound DNA clone in picotitre well, DNA polymerase is added to the well, nucleotides are washed over in turn, deoxynucleoside triphosphate incorporation monitored via pyrophosphate release; Ion Torrent, similar to 454 pyrosequencing, deoxynucleoside triphosphate incorporation monitored via H^+^ ions release detected by a pH sensor that uses complementary metal-oxide-semiconductor technology. SOLiD™, microbead-bound DNA template flanked by adapters is hybridized to a growing complementary strand, under the action of DNA ligase. cPAL, another sequencing by ligation technique not described in this paper, employed by Complete Genomics, anchor sequence and probes hybridize to DNA template in a series of ligation reactions taking place on a nanoball. PacBio single-molecule real-time, sequencing takes place on zero-mode waveguide chip. DNA polymerase at the bottom of the well, fluorescent nucleotides being added to the strand. Oxford nanopore technology relies on changes in ion flow as nucleotides pass through the nanopore.

Pyrosequencing relies on the release of pyrophosphates when nucleotides are washed over fixed DNA clones in a DNA polymerase-mediated reaction (Figure 1). Pyrophosphate takes part in a cascade of reactions that eventually give off light which is detected and interpreted by a computer program. Ion Torrent employs a similar technique except that it uses pH instead of luminescence to detect nucleotide addition, whereas the Applied Biosystems Inc. SOLiD™ system is a sequencing--by-ligation technique which detects fluorescence that results from the action of DNA ligase on oligonucleotides (Figure 1).^5,6^

SOLiD™ and Ion Torrent have been in the NGS market since 2007 and 2010, whereas Roche announced the phaseout of 454 Pyrosequencing in 2013 after facing stiff competition in the market. The reversible terminator sequencing currently sold by Illumina is the predominant NGS platform: a sequencing-by-synthesis approach that uses bridge amplification and fluorescent signals from the addition of deoxynucleoside triphosphate (dNTP) to a growing complementary strand (Figure 1). The signal is detected in real time by a coupled charge device camera and interpreted by computer software.^1^

**FIGURE 2:**
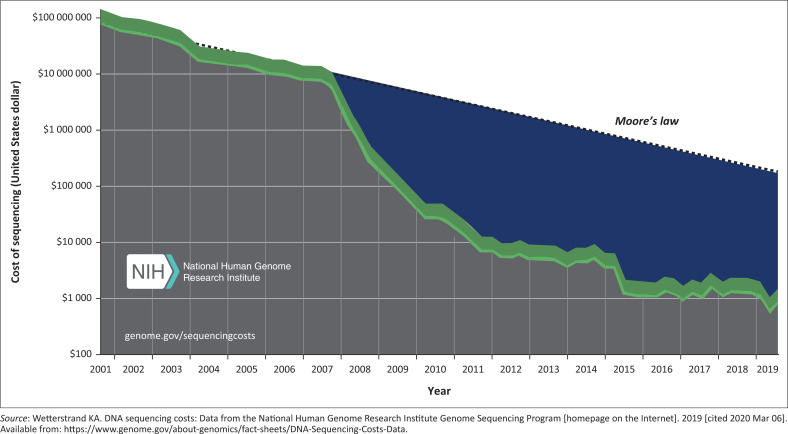
The cost of sequencing a single human genome from 2001 to 2019. The y-axis represents the cost of sequencing a human genome, the x-axis is labelled from year 2001 to 2019. Moore’s Law predicts a biennial doubling of transistors on a microchip. The line drawn in the graph above illustrates Moore’s law prediction of the cost of sequencing per human genome. It is shown clearly that the cost has been consistently lower than predicted by Moore’s Law since 2008.

Next-generation sequencing technologies have made sequencing much easier, faster and cheaper than Sanger sequencing. The August 2019 report from the National Human Genome Research Institute put the cost of sequencing a complete human genome at $942.00 United States dollars (USD). Thanks to NGS, the reduction in cost of genome sequencing has beaten Moore’s Law prediction (Figure 2), more evidently since 2008, about the same time that NGS became popular in sequencing centres.^7^

Illumina, introduced in 2006, is the most robust of all NGS technologies. It quickly gained traction among scientists, because of its high throughput and affordability compared to 454 Pyrosequencing, SOLiD™ and Ion Torrent. In fact, Illumina was the first company to fulfil the ‘$1000 genome’ promise with its HiSeq X Ten sequencer in 2014. Sequencing a genome now costs below $1000.00 USD with ongoing efforts to reach the $100.00 USD target.^3^

## Where short reads come short

All second-generation sequencing technologies have a common limitation – the inability to sequence long stretches of DNA. To sequence a large genome like human DNA using NGS, the DNA has to be fragmented and amplified in clones of between 75 base pairs and 400 base pairs, hence the term ‘short-read sequencing’ (SRS). Computer programs are then used to assemble the random clones into a contiguous sequence.^2^ Aside from the fact that polymerase chain reaction – a necessary step in SRS – causes preferential amplification of repetitive DNA, SRS fails to generate sufficient overlap sequence from the DNA fragments. This constitutes a major challenge for *de novo* sequencing of a highly complex and repetitive genome like the human genome.^2^ The detection of large sequence changes is another area of difficulty that is encountered using SRS – a major barrier to studying structural variations.^8^

## Long-read sequencing

Third-generation sequencing technologies which are otherwise called long-read sequencing (LRS) technologies address the shortcomings of NGS. Whereas the Sanger and SRS approaches cannot exceed read lengths of 1 kilobase pair, third-generation sequencing technologies read lengths are between 5 kilobase pairs and 30 kilobase pairs. The longest read length ever generated by a third-generation sequencing technology is 2 gigabase pairs.^9^ Long-read sequencing methods sequence a single molecule – thus abolishing amplification bias – and generate a reasonable length of overlap sequence for better sequence assembly.^1^

There are two prominent LRS technologies namely, nanopore sequencing and the single-molecule real-time (SMRT) sequencing.^10^ Single-molecule real-time sequencing was commercially released in 2011 by Pacific Biosciences (PacBio). A SMRT genome sequencer consists of millions of zero-mode waveguides – microscopic wells with DNA polymerase fixed at the bottom. Within each zero-mode waveguide, two hairpin adaptors are ligated to both ends of the molecule to form a circular single-stranded DNA. With the aid of a primer complementary to the adaptors, DNA polymerase is used to sequence a complementary strand. When a fluorescent nucleotide is added to the strand, a fluorescence of wavelength corresponding to the added nucleotide is given off. This signal is captured in real time by a coupled charge device camera and interpreted by a computer program.^1^

Nanopore sequencing, released by Oxford Nanopore Technologies in 2014, works by a different principle: threading the DNA molecule through a 1.5 nm wide bioengineered channel embedded in a biological membrane. Electrical current across the channel depends on which nucleotide is traversing the channel at the time. This variation is used to determine the base sequence of the nucleic acid.^1^

Single-molecule real-time and nanopore sequencing check all of the boxes in terms of suitability for *de novo* sequencing, resolution of repeat sequences (e.g. human leukocyte antigen gene, centromere), detection of structural variations, epigenetics and transcriptome sequencing, among others. However, the accuracy per read and the throughput of LRS is poor compared to SRS.^1,10^ Moreover, there are challenges in the adaptability of LRS for sequencing different genome lengths, as LRS data processing takes longer for organisms with large genomes than for viral genomes.^10^

The high error rates of nanopore sequencing are attributed to the poor sensitivity of the nanopore and the inability to control the speed of DNA translocation through the pore. Whereas the errors in nanopore sequencing are systematic, SMRT base-calling errors are random and can be reduced using circular consensus sequencing (CCS), a method that allows the DNA to have multiple passes through the zero-mode waveguides. Increasing the accuracy of SMRT via CCS comes at a higher cost than using NGS.^3^ Nanopore sequencing has improved over the years, although it is still not as accurate as NGS.

## ‘Nano-promises, giga-prospects’

MinION, a small-sized nanopore sequencing device similar to a universal serial bus flash drive, was used to sequence the Ebola virus during the 2014-2016 outbreak in West Africa in 60 min.^3^ The MinION sequencer is much more affordable when compared with Illumina and SMRT considering the cost of instruments and consumables.^9^ Considering its portability, affordability and its ability to generate ultra-long reads, the possibility of using MinION to accurately determine larger genome sequences is of immense value to researchers and clinicians. It holds the answer to affordable genome sequencing in low-resource settings and at the point of care. With improving nanopore technology aimed at decreasing MinION’s high error rates, these promises may not be far-fetched.

The Sequel II 8M SMRT cell, which was launched in 2019 by PacBio, has a CCS read accuracy of 99% and above. PromethION, also released in 2019 by Oxford Nanopore Technologies, generates ultra-long reads, although with less accuracy than SMRT. In their efforts to beef up quality, both companies update their products regularly. The latest -double-sensored nanopore cell R10 and linear consensus sequencing (a similar method to SMRT CCS) are expected to improve the accuracy of Oxford Nanopore Technologies products. Interestingly, LRS technologies are approaching SRS in terms of cost and accuracy. Using the Sequel II 8M SMRT cell that was released, CCS reads can now be obtained for about $1500.00 – an eight-fold reduction in costs.^3^ Less accurate long reads from the PromethION flow cell can be obtained at about the same price.^3,5^ Error rates for LRS used to be 12% – 15%^5^ but have now fallen to less than 1% for SMRT and less than 5% for nanopore sequencers^10^ compared to an error rate of approximately 0.1% in NGS.^3,5^

## Situation report

Notwithstanding SRS market dominance, LRS has broadened its range of applications in recent years as a result of significant improvements in accuracy and throughput. For instance, there has been an increase in the use of MinION in metagenomics studies. LRS technologies are increasingly being used in epigenetics and transcriptomics as well.^10^

The technical difficulties associated with *de novo* assembly of short reads for large complex genomes will likely persist for the foreseeable future. The question then becomes whether LRS technologies will improve on their own shortcomings to take over the market.

A paradigm shift in the prevailing technology is likely to occur if there is a major scientific breakthrough, such as the discovery of a DNA polymerase with a longer lifespan for higher throughput and accuracy in SMRT base calling, or a drastic improvement in the sensitivity and DNA translocation speed in nanopore sequencing that matches the accuracy of SRS technologies. However, such improvements are more likely to take place gradually rather than suddenly; I do not see that occurring in the next 10 years.

## Lessons from the past

History suggests a difficult transition from short-read to long-read technologies. When 454 pyrosequencing was introduced in 2005, it had a clear advantage over Sanger sequencing in terms of throughput and cost. However, funding agencies and scientists were not immediately receptive to the new technology. Many of the researchers had got used to Sanger sequencing, whereas investors were mindful of their stakes. Thus, it took 3 years before NGS gained wide acceptance in the scientific community.^4,7^ Even a major scientific breakthrough in sequencing technologies could still take about 2–3 years before finding its footing in the market for wide acceptability.

As of 2014, Illumina controlled about 70% of the market for DNA sequencers and accounted for 90% of global DNA data.^3^ It is the biggest company on the genomic sequencing market with a worth of $43.6 billion USD. The company’s large and increasing customer base makes it difficult to change sides by motivating customers to buy their stocks.^11^ Currently, no technology compares to Illumina in terms of cost, throughput and accuracy. Even if one were to arise today, it would still have a hard time replacing Illumina, considering the niche that Illumina has carved for itself in the market.

## The Illumina-PacBio merger

The potential of LRS, particularly SMRT, is not unnoticed by market giant Illumina, who entered talks in 2018 on a $1.2 billion USD merger with PacBio. Both parties, however, terminated the deal owing to concerns from the United Kingdom Competition and Markets Authority and the United States Federal Trade Commission about an emerging monopoly.^12^

One is left to imagine why Illumina wanted a merger. Sheer desire for monopoly or the prospect of a robust hybrid technology? Anyway, the synergy of both generations of sequencing technology is worth considering. Hybrid sequencing combines the throughput and accuracy of SRS with the long read length of LRS. It is highly effective for genome polishing^10^ and can produce reference genomes for most organisms.^3^

## Outlook

While it may be difficult to predict with certainty major breakthroughs capable of altering the narrative in genomic sequencing, it is apparent that LRS holds the key to many unanswered questions in genomics. Long-read sequencing technologies, although constantly improving and gradually closing the gap on NGS in terms of accuracy and costs,^3,5^ have yet to attain their full potential. In this decade, I expect that LRS technologies will gain more recognition and acceptance as they are adapted for sequencing a variety of organisms. In the absence of an innovation in LRS technology that can significantly increase accuracy and throughput and reduce costs far beyond what SRS technologies offer, I would argue that it is unlikely that LRS technologies completely replace SRS technologies in the next 10 years. Meanwhile, in the middle of these improvements in SRS and LRS technologies, I expect to see increased efforts towards making hybrid sequencing more scalable.
